# Commensal Fecal Microbiota Profiles Associated with Initial Stages of Intestinal Mucosa Damage: A Pilot Study

**DOI:** 10.3390/cancers16010104

**Published:** 2023-12-24

**Authors:** Sergio Ruiz-Saavedra, Silvia Arboleya, Alicja M. Nogacka, Carmen González del Rey, Adolfo Suárez, Ylenia Diaz, Miguel Gueimonde, Nuria Salazar, Sonia González, Clara G. de los Reyes-Gavilán

**Affiliations:** 1 Department of Microbiology and Biochemistry of Dairy Products, Instituto de Productos Lácteos de Asturias (IPLA-CSIC), 33300 Villaviciosa, Spain; sergio.ruiz@ipla.csic.es (S.R.-S.); silvia.arboleya@ipla.csic.es (S.A.); alicja.nogacka@ipla.csic.es (A.M.N.); mgueimonde@ipla.csic.es (M.G.); nuriasg@ipla.csic.es (N.S.); 2 Diet, Microbiota and Health Group, Instituto de Investigación Sanitaria del Principado de Asturias (ISPA), 33011 Oviedo, Spain; adolfo.suarez@sespa.es; 3 Department of Anatomical Pathology, Central University Hospital of Asturias (HUCA), 33011 Oviedo, Spain; carmenchugonzalezdelrey@gmail.com; 4 Digestive Service, Central University Hospital of Asturias (HUCA), 33011 Oviedo, Spain; 5 Digestive Service, Carmen and Severo Ochoa Hospital, 33819 Cangas del Narcea, Spain; yleniads@hotmail.com; 6 Department of Functional Biology, University of Oviedo, 33006 Oviedo, Spain

**Keywords:** fecal microbiota, intestinal mucosa, intestinal polyps, hyperplastic polyps, conventional adenomas

## Abstract

**Simple Summary:**

The high incidence and mortality of colorectal cancer (CRC) have influenced society to promote research in this field. Dysbiosis of the intestinal microbiota occurring in CRC has been extensively studied. However, microbial shifts occurring at the initial stages of mucosal alterations are less known. In this work, the fecal microbiota of volunteers diagnosed with intestinal polyps were compared with the microbial compositions of nonpathological control volunteers, thereby focusing on the nature of the hyperplastic polyps or conventional adenomas, as well as on the degree of dysplasia (low grade vs. high grade) in the last ones as indicators of colorectal cancer risk development. The findings provide insights into the microbiota changes occurring at the early stages of intestinal mucosal lesions. This work could set a starting point for further studies focusing on the influence of diet and lifestyle factors on the initial alterations of the intestinal mucosa and for the proposal of strategies for their prevention.

**Abstract:**

Progressive intestinal mucosal damage occurs over years prior to colorectal cancer (CRC) development. The endoscopic screening of polyps and histopathological examination are used clinically to determine the risk and progression of mucosal lesions. We analyzed fecal microbiota compositions using 16S rRNA gene-based metataxonomic analyses and the levels of short-chain fatty acids (SCFAs) using gas chromatography in volunteers undergoing colonoscopy and histopathological analyses to determine the microbiota shifts occurring at the early stages of intestinal mucosa alterations. The results were compared between diagnosis groups (nonpathological controls and polyps), between samples from individuals with hyperplastic polyps or conventional adenomas, and between grades of dysplasia in conventional adenomas. Some microbial taxa from the *Bacillota* and *Euryarchaeota* phyla were the most affected when comparing the diagnosis and histopathological groups. Deeper microbiota alterations were found in the conventional adenomas than in the hyperplastic polyps. The *Ruminococcus torques* group was enriched in both the hyperplastic polyps and conventional adenomas, whereas the family *Eggerthellaceae* was enriched only in the hyperplastic polyps. The abundance of *Prevotellaceae*, *Oscillospiraceae*, *Methanobacteriaceae*, *Streptococcaceae*, *Christensenellaceae*, *Erysipelotrichaceae*, and *Clostridiaceae* shifted in conventional adenomas depending on the grade of dysplasia, without affecting the major SCFAs. Our results suggest a reorganization of microbial consortia involved in gut fermentative processes.

## 1. Introduction

The progression of colorectal cancer (CRC) is a gradual process in which the intestinal mucosa damage generally occurs over years [1,2] and seems to be strongly linked to modifiable external factors, such as lifestyle and diet [3,4,5,6]. The detection of endoscopic polyp lesions, accompanied by the further histological examination of resected polyps or mucosa samples during colonoscopy, is used in routine clinical practice to determine the degree of mucosal damage and the risk of CRC-related lesions [7,8]. Most CRC cases (>80%) arise from the adenomatous pathway of genetic alterations related to phenotypic changes in the adenoma–carcinoma sequence. Conventional colorectal adenomas frequently carry dysplastic cells with low-grade dysplasia (LGD), which generally occurs in tubular adenomas (TAs), tubulovillous adenomas (TVAs), or with high-grade dysplasia (HGD). The alternative pathway is less frequent (<20%), originates from *BRAF* and *KRAS* gene mutations, and is linked to the progression of serrated lesions to carcinoma. The serrated lesions include hyperplastic polyps (HPs), as well as traditional serrated adenoma and sessile serrated lesions (SSLs) that normally contain cells with dysplasia [9]. Hyperplastic polyps generally present low risk of evolving to neoplasia, whereas LGD and HGD present a progressively augmented risk of adenocarcinoma development [7,10,11]. 

Changes occurring over time on the intestinal microbiota, which are partly driven by their interaction with dietary components, can influence host health [12,13,14]. Dietary components, such as complex carbohydrates (fibers) and, to a lesser extent, peptides and fats, reach the colon undigested and can be used as fermentable substrates by the colonic microbiota. The major microbial products of these fermentations are short-chain fatty acids (SCFAs), which mainly include acetate, propionate, and butyrate, that promote differential effects in host health [12,13]. In contrast, the ammonium, phenolic, and indolic compounds produced by the microbial colonic fermentation of aromatic amino acids, as well as hydrogen sulfide, have mutagenic, genotoxic, and cytotoxic potential regarding the intestinal mucosa, which could alter the microbiota [14]. Some intestinal microorganisms can produce and excrete toxic compounds as toxins and some protease-like factors, such as the colibactin produced by *Escherichia coli*, the cytotoxin A produced by *Helicobacter pylori*, an enterotoxin produced by *Bacteroides fragilis*, the adhesin A produced by *Fusobacterium nucleatum*, or the cysteine protease-like factor produced by *Shigella flexneri*, among others [15]. All these factors modify the intestinal environment and may contribute to the onset and progression of gut mucosal damage, as well as the further establishment of preneoplastic lesions.

Dysbiosis occurs in CRC and in preneoplastic stages. The “driver-passenger” theory tries to explain how the gut bacteria can induce carcinogenesis through progressive damage of the DNA in the gut epithelium (“drivers”), thereby modifying the gut environment to promote the proliferation of “passenger” bacteria [16]. Most studies have focused on the microbiota of CRC, whereas less information is available regarding shifts in the microbiota profiles in the initial intestinal mucosal lesions that develop prior to CRC [17,18]. This pilot study sheds light on the changes to the fecal microbiota associated with early intestinal mucosal lesions by considering the morphological alterations of intestinal polyps (hyperplastic and conventional adenomas) and the grade of dysplasia in the adenomatous pathway of carcinogenesis. 

## 2. Materials and Methods

### 2.1. Study Design and Volunteers

Volunteers were randomly recruited by trained physicians from among those subjects who reached out to the Digestive Section at the Central University Hospital of Asturias (HUCA) or to the Carmen and Severo Ochoa Hospital in Asturias (North of Spain) for consultation about clinical symptoms or as part of the CRC screening program in the region between October 2019 and December 2021. To enroll in the study, volunteers aged 40–79 years were first informed about the objectives and procedures and signed an informed consent to participate. Anamnesis was performed, and fecal samples were collected at the time of recruitment. Biopsies of the intestinal mucosa and polyps were resected during colonoscopy and examined at the Department of Anatomical Pathology of HUCA, as described elsewhere [19]. Patients reporting specific cancer treatment at the time of the study, treatment with medical drugs in the previous two months, immune-related diseases, or previous surgery of the digestive system were excluded. Individuals were classified according to the colonoscopy and histopathological examination. The group of “controls” included individuals with normal colonoscopy results and confirmation of the absence of mucosal lesions according to histopathological analysis, whereas the group of “polyps” included those individuals with alterations detected through colonoscopy results and confirmed by histopathological analyses. This group included individuals with either hyperplastic polyps or conventional adenomas as per morphological features detected by histopathology; conventional adenomas were subclassified depending on the grade of dysplasia. This project was evaluated and approved by the Regional Ethics Committee of the Clinical Research of Asturias (Ref. 163/19) and the Committee on Bioethics of CSIC (Ref. 174/2020). This study followed the fundamental principles of the Declaration of Helsinki, through the Council of Europe Convention on Human Rights and Biomedicine, and Spanish legislation on bioethics. Personal data protection was treated according to Directive 95/46/EC of the European Parliament and the Council of October 1995 on the Protection of Individuals.

### 2.2. Fecal Sample Processing

Instructions and material necessary for collecting fecal samples were provided to the volunteers. Briefly, patients deposited fresh fecal samples in sterile plastic containers and transported them to the hospitals participating in the study within a period not exceeding two hours after deposition. The samples were then frozen and transported to the laboratory. Frozen samples (4 g) were weighed, diluted 1/10, and homogenized with sterile phosphate-buffered saline (PBS) in a LabBlender 400 Stomacher (Seward Medical, London, UK) for 3 min at maximum speed. After 15 min of centrifugation at 4 °C and 14,000 rpm, the supernatants and pellets were separated and kept frozen at −20 °C until use.

### 2.3. DNA Extraction and Microbiota Metataxonomic Analyses

The fecal pellets obtained after dilution and homogenization were used to extract DNA. The Q protocol for DNA extraction defined by the International Human Microbiome Standards Consortium was applied using the QIAamp Fast DNA Stool Mini Kit (Qiagen, Sussex, UK) [20]. After extraction, a 260/280 ratio was determined using a Take3 Microvolume plate and Gen5 microplate reader (BioTek Instrument Inc., Winooski, VT, USA). DNA was frozen at −20 °C until analysis. Sequencing and annotation of the bacterial 16S rRNA genes were performed at Novogene Bioinformatics Technology Co., Ltd., Cambridge, UK. First, using specific primers connected with barcodes, the variable region V3–V4 of bacterial 16S rRNA genes was amplified using PCR protocol, and a DNA library was prepared. The Illumina NovaSeq 6000 platform was used to sequence the libraries. After obtaining each individual read, they were assigned to the samples using barcodes and merged using FLASH (version 1.2.7). QIIME (version 1.7.0) was used to obtain high-quality clean tags, thereby allowing the removal of low-quality sequences. The obtained tags were then compared with the reference SILVA 138 database, and chimeric sequences were removed using the UCHIME algorithm. To perform sequencing analysis, effective tags were utilized using Uparse software (Uparse V 7.0.1090). Sequences sharing ≥97% homology were assigned to the same OTUs, and OTU abundance was normalized. The representative sequence for each OTU was obtained against the SSU rRNA database of SILVA138 using QIIME (V 1.7.0) and the mothur method to annotate species at each taxonomic rank. Chao1 and Shannon indices of alpha diversity were obtained using QIIME (V 1.7.0) and are represented using GraphPad Prism 9 software. PCoA of beta diversity was calculated based on Bray Curtis dissimilarity index and visualized using “vegan” and “ggplot2” packages of RStudio software version 1.4.3.

### 2.4. Fecal Short-Chain Fatty Acid Concentrations

Gas chromatography analyses were performed to determine the concentration of SCFAs, acetic, propionic, butyric, isobutyric, isovaleric, valeric, and caproic acids, in feces following a previously described procedure, with minor modifications [21]. Briefly, fecal supernatants were diluted with methanol, 20% *v*/*v* formic acid, and an internal standard to reach a dilution of 10/65. Next, the dilutions were centrifuged for 10 min at room temperature and 14,000 rpm to obtain the supernatants, which were transferred to suitable chromatography vials. To identify and quantify SCFAs, a chromatograph 6890N (Agilent Technologies Inc., Palo Alto, CA, USA) connected to a mass spectrometry detector (MS) 5973N (Agilent Technologies) and a flame ionization detector (FID) were used. The theoretical detection limit values were calculated for minor SCFAs and applied to samples that were not detectable.

### 2.5. Statistical Analyses

IBM SPSS software (version 25.0; IBM SPSS, Inc., Chicago, IL, USA) and RStudio software version 1.4.3 were used to analyze the data. GraphPad Prism 9 and RStudio software were used for graphical representations. Goodness of fit to the normal distribution was checked using the Kolmogorov–Smirnov test. As normality of the variables was not achieved, nonparametric tests were applied. Overall, categorical variables were presented as numbers and percentages, and continuous variables were presented as mean ± standard deviation values. Mann–Whitney U tests were performed for pairwise comparison of continuous variables (*p*-value < 0.05), whereas the categorical variable “gender” was analyzed by a chi-squared test. 

Alpha and beta diversity indices were compared between diagnosis groups. A Mann–Whitney U test was applied to compare alpha diversity indices, whereas PCoA was performed on beta diversity using Bray–Curtis dissimilarity index. Relative microbial abundances were obtained from OTU data and analyzed using Mann–Whitney U tests to detect differences between groups at the taxonomic levels of phylum, family, and genus. The Benjamini–Hochberg procedure was applied for multiple test comparisons. LEfSe were conducted to estimate taxa by significantly discriminating the groups under study by using the Kruskal–Wallis rank sum test and a Wilcoxon test for pairwise comparison followed by a logarithmic linear discriminant analysis (LDA) to estimate the effect size at a threshold of 2.0, by using the LEfSe Galaxy web tool [22]. Only microbial families and genera with relative abundance of at least 1% in at least two samples were considered in the analysis. The differences in SCFA concentrations between the groups were compared using the Mann–Whitney test. Spearman correlations were performed to explore the associations between microbiota and SCFAs. Heatmaps were generated using “corrplot” R package. Microbial families significantly correlated with SCFAs were examined as predictors of fecal SCFAs using regression analyses, adjusting by diagnosis, and histopathological groups. 

## 3. Results

### 3.1. General Characteristics of the Sample Population

The main anthropometric characteristics of volunteers from each diagnosis group and the main histopathological features of mucosal biopsies are shown in Table 1. Individuals in the control group did not present histological lesions of the intestinal mucosa, while volunteers with polyps displayed hyperplastic polyps or conventional adenomas. Individuals presenting conventional adenomas were more abundant than those with hyperplastic polyps (25 vs. 9 individuals). Conventional adenomas presented LGD (TA or TVA polyps) or HGD. 

### 3.2. Fecal Microbiota Profiles

Stool microbiota compositions were determined using partial 16S rRNA gene sequencing. The 54 fecal samples yielded 9585 OTUs. We compared the microbiota profiles according to diagnosis and histopathological groups (nonpathological controls and polyps: 20 and 34 samples, respectively) and analyzed the differences in microbiota between individuals presenting hyperplastic polyps (nine samples) or conventional adenomas (25 samples) within the group of polyps. We further analyzed the fecal microbiota of individuals carrying conventional adenomas with LGD (20 samples) or HGD (five samples).

#### 3.2.1. Microbiota Comparison between Control and Polyps Groups

**Figure 1:**
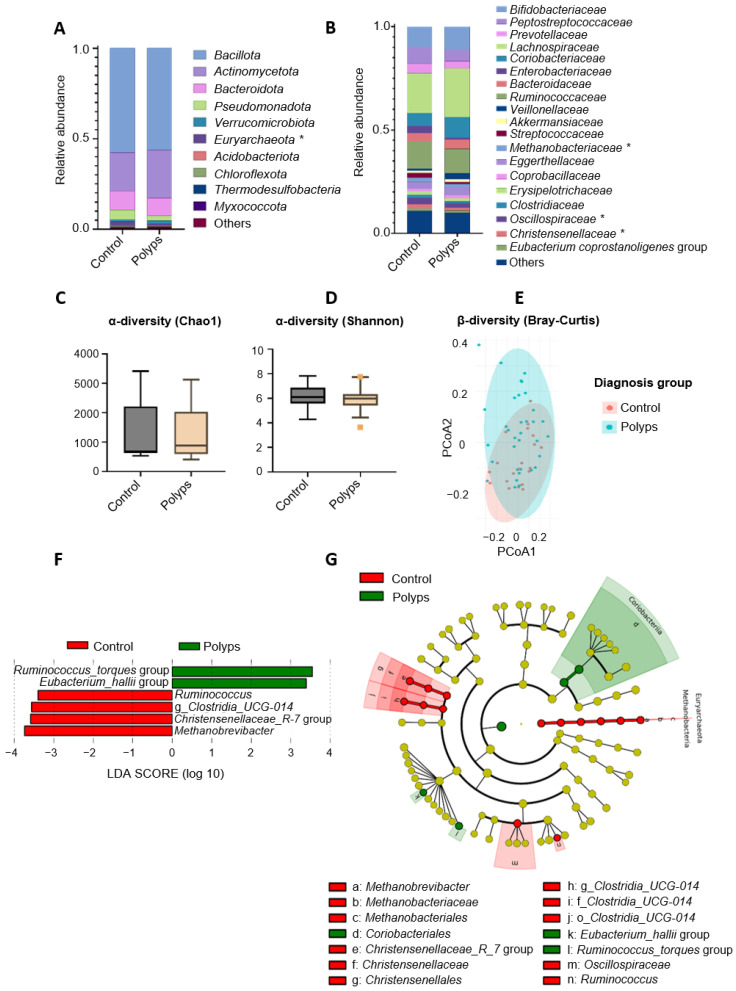
Differences in composition and taxonomic diversity of fecal microbiota between diagnosis groups (control and polyps). (**A**) Relative abundance at the phylum level (including top-10 most abundant). (**B**) Relative abundance of bacteria at the family level (including families showing relative abundance >1% in the sample population). *, Statistically significant differences between control and polyps groups (Mann–Withney U test adjusted by Benjamini–Hochberg; *p* < 0.05). Box plots of alpha diversity values: (**C**) Chao1 index and (**D**) Shannon index. Box elements show the median, upper, and lower quartiles. Dots represent values outside the IQR. (**E**) PCoA of beta diversity using Bray–Curtis dissimilarity index. (**F**) Bacterial taxa (at the genus taxonomic level) showing differential abundance according to linear effect size discriminant analysis (LEfSe) for the comparison of control vs. polyps groups. (**G**) Cladogram generated from LEfSe between control and polyps groups. Yellow circles represent bacterial taxa that show no significant differences between groups, and green and red circles represent taxa whose abundances were statistically differential between groups. For LEfSe, only genera with relative abundances higher than 1% in at least two samples were included.

Figure 1 presents the main fecal microbiota profiles of the two groups: the control and polyps. The relative abundance of phyla in the control and polyps groups followed the order *Bacillota* (former *Firmicutes*) > *Actinomycetota* > *Bacteroidota* > *Pseudomonadota* (formerly *Proteobacteria*) > *Verrucomicrobiota* (Figure 1A). The family *Methanobacteriaceae* was significantly less abundant in the polyps group than in the control group (1.37% vs. 2.11%, respectively), and the same was observed for *Oscillospiraceae* and *Christensenellaceae*, which were more abundant in the control group than in the polyps group (Figure 1B) (3.08% vs. 2.18% and 1.89% vs. 1.26%, respectively). Alpha diversity, represented by Chao1 and Shannon indices (Figure 1C,D), did not differ significantly between the groups. Regarding beta diversity, principal coordinate analysis (PCoA) based on the Bray–Curtis dissimilarity index using all OTU normalized relative abundances did not clearly separate the groups (Figure 1E). Linear discriminant analysis effect size (LEfSe) testing was conducted to identify the genera contributing the most to differentiating the control and polyps group. Differentially increased abundances of the *Ruminococcus_torques* group (family: *Lachnospiraceae*; order: *Lachnospirales*) and *Eubacterium_hallii* (family: *Lachnospiraceae*; order: *Lachnospirales*) in contrast to decreased abundances of *Ruminococcus* (family: *Ruminococcaceae*; order: *Oscillospirales*), g_*Clostridia_UCG-014*, the *Christensenellaceae R-7* group (family: *Christensenellaceae*; order: *Chistensenellales*), and *Methanobrevibacter* (family: *Methanobacteriaceae*; order: *Methanobacteriales*) were the most relevant differences found between the feces samples from individuals diagnosed with polyps with respect to the controls (Figure 1F). The cladogram generated from the LEfSe results (Figure 1 G) highlights the enrichment of the order *Coriobacteriales* and members from the family *Lachnospiraceae*, as well as the depletion of the families *Christensenellaceae*, *Oscillospiraceae*, f_*Clostridia_UCG-014*, and *Methanobacteriaceae* in the polyps group. 

#### 3.2.2. Microbiota Comparison between Individuals with Hyperplastic Polyps and Conventional Adenomas

**Figure 2:**
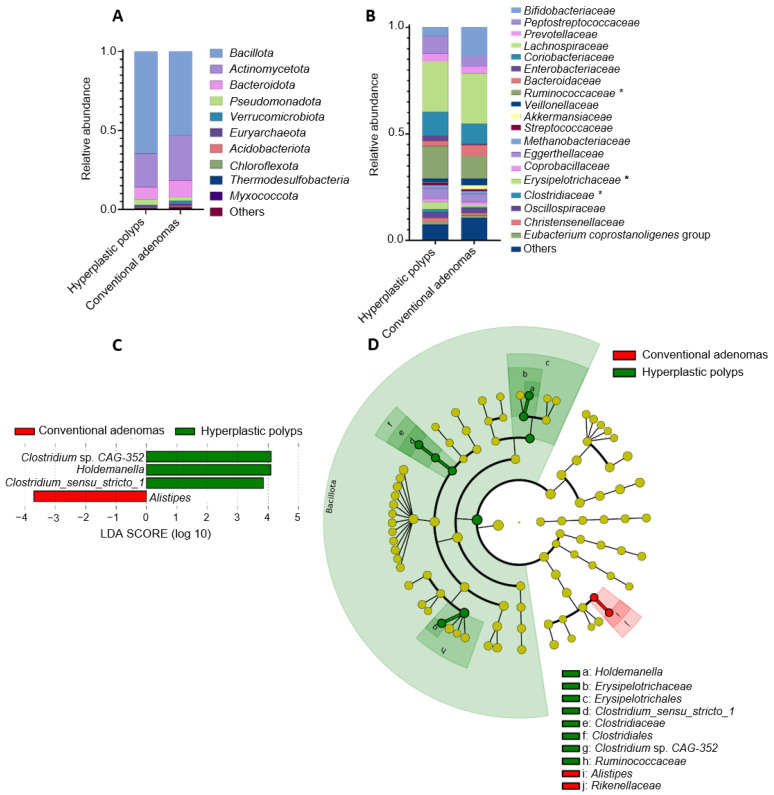
Differences in fecal microbiota compositions from individuals diagnosed with hyperplastic polyps or conventional adenomas. (**A**) Relative abundance of bacteria at the phylum level (including top-10 most abundant). (**B**) Relative abundance of bacteria at the family level (including those families showing relative abundance >1% in the sample). *, Statistically significant differences between samples from individuals with hyperplastic polyps or conventional adenomas (Mann–Whitney U test adjusted by Benjamini–Hochberg; *p* < 0.05). (**C**) Bacterial taxa (at the genus taxonomic level) showing differential abundance according to linear effect size discriminant analysis (LEfSe) for the comparison of hyperplastic polyps and conventional adenomas groups. (**D**) Cladogram generated from LEfSe between samples from individuals diagnosed with hyperplastic polyps or conventional adenomas. Yellow circles represent bacterial taxa that show no significant differences between groups, and green and red circles represent taxa whose abundances were significantly differential between groups.

We compared the fecal microbiota of individuals with hyperplastic polyps and those with conventional adenomas (Table 1). Differences in the fecal microbiota profiles were observed at phylum and family taxonomic level (Figure 2A,B). Significantly (*p* < 0.05) lower abundances of the *Ruminococcaceae*, *Erysipelotrichaceae*, and *Clostridiaceae* families were found in fecal samples from individuals with conventional adenomas than in those presenting hyperplastic polyps (Figure 2B) (10.70% vs. 15.22%, 0.88% vs. 3.52% and 0.80% vs. 1.37%, respectively). LEfSe analysis (Figure 2C) evidenced that the genera *Holdemanella* (family: *Erysipelotrichaceae*; order: *Erysipelotrichales*), *Clostridium sensu_stricto 1* (family: *Clostridiaceae*; order: *Clostridiales*), and *Clostridium* sp. *CAG-352* (family *Ruminococcaceae*; order: *Oscilllospirales*) were enriched in the feces of individuals with hyperplastic polyps, which was in contrast to the higher comparative abundance of *Alistipes* (family *Rikenellaceae*; order: *Bacteroidales*) in the group of individuals with conventional adenomas. Cladograms generated from the LEfSe (Figure 2D) confirmed differential abundances for the upper taxa related to the genera mentioned above, including the *Erysipelotrichaceae*, *Clostridiaceae*, and *Ruminococcaceae* families, thereby also affecting the orders *Erysipelotrichales* and *Clostridiales*, as well as the *Bacillota* phylum. 

#### 3.2.3. Shifts in the Microbiota of Individuals with Hyperplastic Polyps

**Figure 3:**
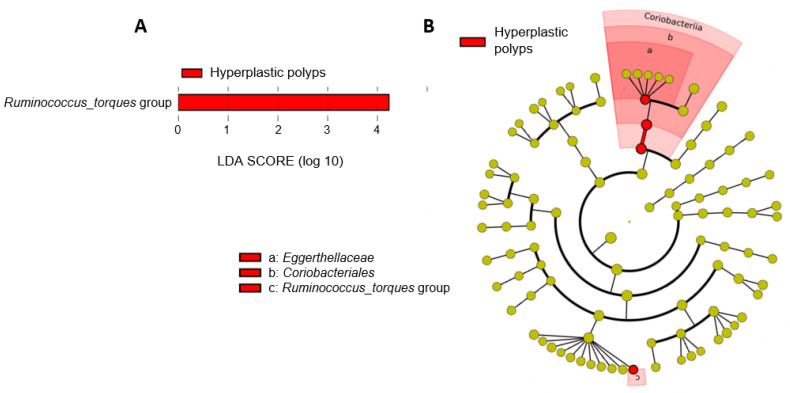
Differences in fecal microbiota compositions from individuals diagnosed with hyperplastic polyps compared to control samples. (**A**) Microbial taxa (at the genus taxonomic level) showing differential abundances according to linear effect size discriminant analysis (LEfSe) for the comparison of samples from individuals diagnosed with hyperplastic polyps and individuals from the control group. (**B**) Cladogram generated from LEfSe between samples from individuals with hyperplastic polyps and the control group. Yellow circles represent bacterial taxa showing no significant differences between groups, and red circles represent taxa that were significantly more abundant in the group with hyperplastic polyps.

To identify changes that specifically affected the microbiota of individuals with hyperplastic polyps, we compared their fecal microbiota with that from the control group (Figure 3). LEfSe analysis at the genus level revealed a differentially higher abundance of *Ruminococcus_torques* in the feces of individuals with hyperplastic polyps as compared to the controls (Figure 3A). The corresponding cladogram also showed a differentially higher abundance of the family *Eggerthellaceae* and the order *Coriobacteriales* in the group with hyperplastic polyps (Figure 3B).

#### 3.2.4. Shifts in the Microbiota of Individuals with Conventional Adenomas

**Figure 4:**
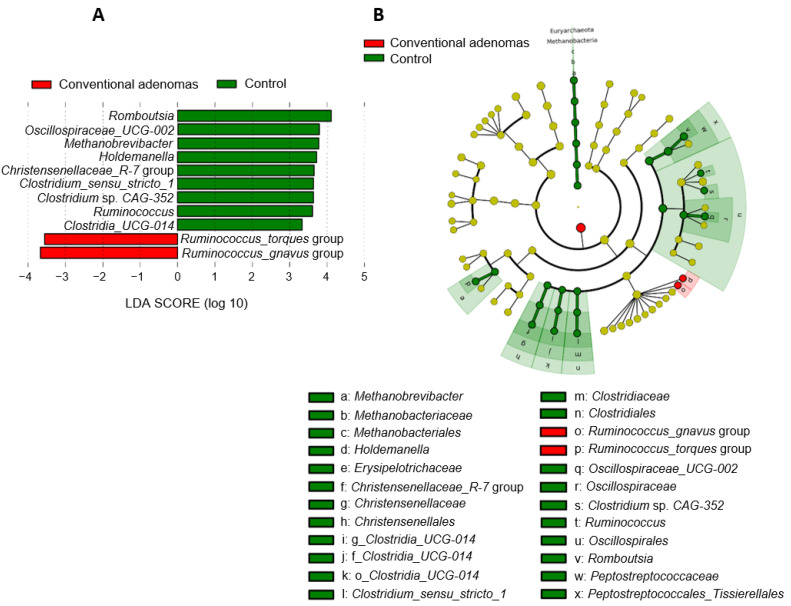
Differences in fecal microbiota compositions from individuals diagnosed with conventional adenomas compared with control samples. (**A**) Microbial taxa (at the genus taxonomic level) showing differential abundance according to linear effect size discriminant analysis (LEfSe) for the comparison of samples from individuals with conventional adenomas and individuals from the control group. (**B**) Cladogram generated from LEfSe between samples from individuals with conventional adenomas and the control group. Yellow circles represent bacterial taxa showing no significant differences between groups, and red circles represent taxa that were significantly more abundant in the group with conventional adenomas.

The fecal microbiota of individuals diagnosed with conventional adenomas were compared with the microbiota of the control group. We further compared the microbiota from individuals without intestinal mucosal lesions and the microbiota from those presenting conventional adenomas with different grades of dysplasia (LGD and HGD). LEfSe analysis showed a differential enrichment of the *Ruminococcus_torques* and *Ruminococcus_gnavus* groups in patients with conventional adenomas (Figure 4A,B). In contrast, a differential depletion of the following taxa was also found in these individuals: genus *Methanobrevibacter*/family *Methanobacteriaceae*/order *Methanobacteriales*; genus *Holdemanella*/family *Erysipelotrichaceae*; genus *Christensenellaceae R-7* group/family *Christensenellaceae*/order *Christensenellales*; genus *Clostridia_UCG-014* and its related upper taxa; the *Clostridium_sensu_stricto_1*/family *Clostridiaceae*/order *Clostridiales*; genus *Ruminococcus*, *Oscillospiraceae_UCG-002*, and *Clostridium* sp. *CAG-352* within the order *Oscillospirales*; and genus *Romboutsia*/family *Peptostreptococcaceae*/order *Peptostreptococcales*_*Tissierellales*. 

**Figure 5:**
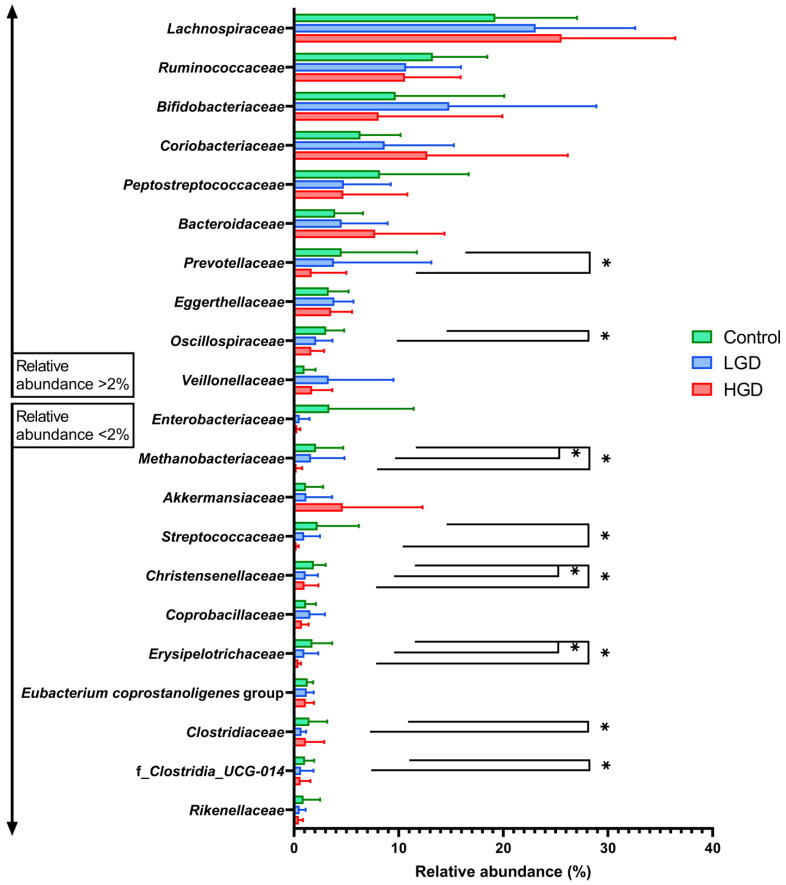
Relative abundances of the main microbial families according to the grade of dysplasia in individuals with conventional adenomas. (*) Statistically significant differences between LGD or HGD with respect to control are indicated by linked upper-black bars (Mann–Whitney U test adjusted by Benjamini–Hochberg; *p* < 0.05). LGD: low-grade dysplasia; HGD: high-grade dysplasia.

The relative abundance of the phylum *Euryarchaeota* decreased abruptly in the feces of individuals with conventional adenomas displaying HGD as compared to control samples (Table S1). A consistent decrease (*p* < 0.05) related to the increase in the grade of dysplasia was found for the families *Methanobacteriaceae* (2.11% in controls; 1.63% in LGD; 0.26% in HGD), *Christensenellaceae* (1.89% in controls; 1.11% in LGD; 1.00% in HGD), and *Erysipelotrichaceae* (1.75% in controls; 0.99% in LGD; 1.00% in HGD) (Figure 5). Notably, the families *Oscillospiraceae*, *Clostridiaceae*, and f_*Clostridia*_UCG-014 showed a significant decrease in samples from individuals with LGD conventional adenomas with respect to the control group (2.12% vs. 3.08 %, 0.72% vs. 1.47% and 0.70% vs. 1.05%, respectively), whereas *Prevotellaceae* and *Streptococcaceae* decreased significantly in samples from volunteers with HGD conventional adenomas (1.68% vs. 4.55% and 0.25% vs. 2.25%, respectively). Significant variations in some microbial genera from the families mentioned just above as related to the grade of dysplastic lesions supported these results: these included *Clostridium_sensu_stricto_1* (*Clostridiaceae*), *Streptococcus* (*Streptococcaceae*), *Methanobrevibacter* (*Methanobacteriaceae*), *Holdemanella* (*Erysipelotrichaceae*), *Christensenellaceae_R-7 group* (*Christensenellaceae*), and genus *Clostridia_UCG-014* (Table S2).

### 3.3. Fecal Short-Chain Fatty Acids

As expected, acetic acid was the major fecal SCFA, followed by propionic and butyric acid, in the two diagnosis groups: the control and polyps (Table S3). Isobutyric, isovaleric, valeric, and caproic acids were present in considerably low proportions. Decreased levels of caproic acid in the polyps group with respect to the control group was the only statistically significant difference found when the SCFA levels were compared according to diagnosis groups. Using histopathological examination as the classification criteria, significantly lower fecal levels of caproic acid were found in samples from individuals with hyperplastic polyps and conventional adenomas when compared with samples from the control group (Table 2).

**Figure 6:**
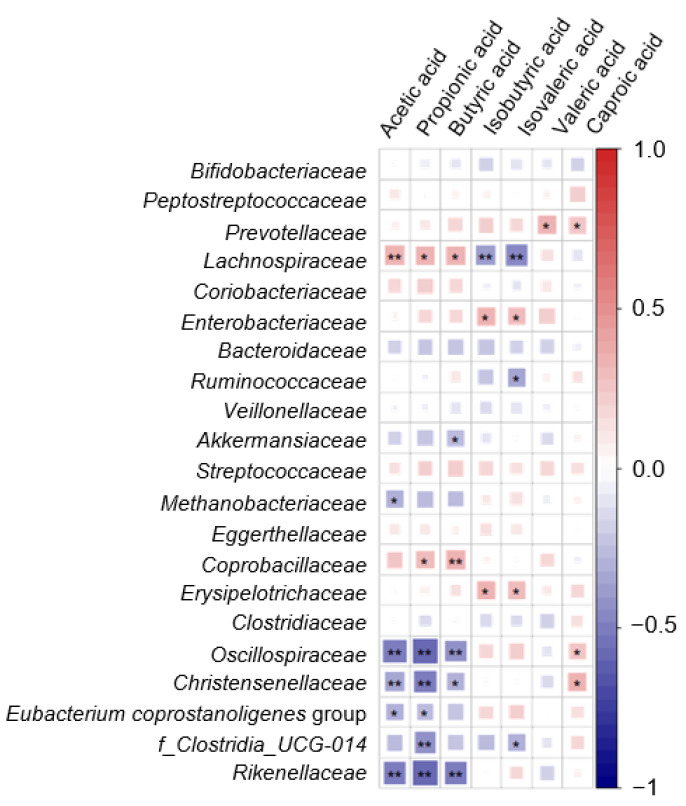
Heatmap defined by Spearman correlations in the sample population under study between most abundant microbial families (relative abundance ≥ 1%) and SCFAs. The intensity of red and blue colors represents the degree of positive and negative associations, respectively. (*) *p* < 0.05; (**) *p* < 0.01.

Correlations between the SCFAs and microbiota evidenced significant positive associations of the *Lachnospiraceae* family with the three major SCFAs and of the *Coprobacillaceae* family with propionic and butyric acid. Negative associations were found between *Oscillospiraceae*, *Christensenellaceae*, and *Rikenellaceae* and the major SCFAs (Figure 6). Significant positive associations were found for *Prevotellaceae*, *Oscillospiraceae*, and *Christensenellaceae* with caproic acid. 

Therefore, to more precisely characterize those microbial families that are potential predictors of the fecal levels of SCFAs, thereby avoiding possible confounding factors, linear regression analyses were conducted through adjustment according to the diagnosis and histopathological groups for those associations displaying significant correlations in Figure 6 (Table 3). The *Oscillospiraceae* and *Rikenellaceae* families were negative predictors of acetic, propionic, and butyric acid in several classification groups, whereas *Coprobacillaceae* was a positive predictor of propionic acid in the control group and of propionic and butyric acid in the group with conventional adenomas. *Lachnospiraceae* was directly associated with acetic acid concentrations only in the control group. *Enterobacteriaceae* was directly associated with the fecal levels of isobutyric and isovaleric acid in several diagnosis and histopathological groups. The *Oscillospiraceae* family was associated with caproic acid in the group with conventional adenomas with HGD. 

## 4. Discussion

Gut microbiota composition is considered as one of the main factors influencing the pathogenesis of CRC. Although alterations have been reported in the fecal microbiota compositions of patients with CRC, the initial shifts in the microbiota associated with early intestinal mucosa damage prior to CRC development are less known [23,24]. In this study, changes to the intestinal microbiota at the initial stages of intestinal mucosal damage were examined according to several criteria: diagnosis groups (control and polyps), morphological type of polyp (hyperplastic vs. conventional adenoma), and grade of dysplasia (low or high) in conventional adenomas. 

In contrast to previous reports, no differences were found in the alpha and beta diversity of fecal microbiota between the diagnosis groups. This discrepancy could be due to the different stages of mucosal damage used for the analysis of the microbiota compositions among studies [25,26,27]. 

The results of the present work indicate that specific and different microbial taxa from the *Bacillota* and *Euryarchaeota* phyla were predominantly affected in the two diagnosis groups: the control and polyps. Genera determining differences between the control and polyps groups included *Ruminococcus*, g_*Clostridia_UCG-014*, the *Christensenellaceae_R-7* group, and *Methanobrevibacter*, which were less abundant in individuals with polyps, and the genera *Eubacterium_hallii* and *Ruminococcus_torques*, which were enriched in the polyps group. The altered abundances of these microorganisms and/or some of the directly related taxa have been reported previously with regard to CRC and preneoplastic stages in the same direction found in the present work [24,25,26,27]. The family *Christensenellaceae*, which was significantly less abundant in the polyps group, has been generally associated with a positive impact on human health, as well as some members of the butyrate-producing family *Ruminococcaceae*, which have a protective effect against the development of CRC that is linked to the consumption of a high-fiber diet and consequent microbial colonic fermentation [28,29,30]. Regarding *Oscillospiraceae*, which also decreased in the polyps group, previous studies have reported a higher abundance of this family in samples of healthy controls or in samples from individuals at the initial stages of CRC as compared to individuals with more advanced CRC [31,32]. Methanogenic archaea in the colon are mostly represented by the genus *Methanobrevibacter*. These microorganisms are functionally associated with the fermentation of dietary fibers through the use of hydrogen produced by saccharolytic bacteria as an electron donor for reduction reactions to produce methane. This contributes to maintain a beneficial circle that provides energy substrates to the host, enhances the epithelial barrier, and protects against intestinal pathogens [33]. However, the imbalance of methanogenic bacteria during dysbiosis, as evidenced in individuals with polyps in the present work, is associated with several dysimmune conditions and CRC [34]. Under normal conditions, *Eubacterium hallii* contributes to the formation of butyrate and propionate in the intestine and has also shown the ability to conjugate xenobiotic compounds such as the heterocyclic amine 2-amino-1-methyl-6-phenylimidazo[4,5-b]pyridine (PhIP), thereby contributing to their detoxification in the colon [35,36]. Moreover, *Eubacterium hallii* was included in a panel of 10 species that were used for differentiating adenomas among CRC patients [37].

Significant differences in the intestinal microbiota compositions have been obtained, thereby affecting some important taxa from the *Bacillota* (including the families *Erysipelotrichaceae*, *Clostridiaceae*, and *Ruminococcaceae*) and *Bacteroidota* phyla, between samples from individuals with hyperplastic polyps and those with conventional adenomas. Mori et al. recently reported alterations in the bacterial communities belonging to the *Firmicutes* phylum (renamed *Bacillota*) in patients with intestinal preneoplastic lesions, including samples from the “adenoma-carcinoma” sequence and hyperplastic polyps [38]. The *Erysipelotrichaceae* family has been considered as a possible indicator of the intermediate steps in the sequence of preneoplastic intestinal mucosal lesions in sporadic carcinogenesis, and it has been related to inflammatory processes of the gastrointestinal tract, as well as to host lipid metabolism [17,38,39]. Among the families whose abundances were found to significantly reduced in individuals with conventional adenomas with respect to hyperplastic polyps, *Ruminococcaceae* is of special relevance, as it is the sole family accounting for a relative abundance over 10% [29].

The results obtained support recent studies pointing to some differences In the intestinal microbiota between the serrated and the conventional adenomatous pathways of carcinogenesis [17,40]. Microbial taxa were found to be more altered in conventional adenomas than in hyperplastic polyps. Remarkably, the *Ruminococcus_torques* group was the only shared taxon that was differentially enriched in samples from individuals with either hyperplastic polyps or conventional adenomas. Members of the *Ruminococcus torques* group have been positively associated with proinflammatory diets and, as also occurs with *Ruminococcus gnavus* (both were differentially enriched in the group with conventional adenomas), were found to be increased in several inflammatory processes and diseases [41,42,43]. *Ruminococcus torques* has been recently identified as a “driver” in the initiation and progression of the cancerous process [44]. 

When the fecal microbiota of individuals with LGD and HGD conventional adenomas were compared, a reduction in the relative abundances of the *Christensenellaceae*, *Methanobacteriaceae*, and *Erysipelotrichaceae* families was obtained as related to the increase in the grade of dysplasia. Mori et al. proposed the *Lachnospiraceae* and *Erysipelotrichaceae* families and members of the phylum *Actinomycetota* as markers of the gut microbiota of healthy individuals and those with low-risk intestinal polyps [38]. The family *Prevotellaceae* was also decreased in individuals with HGD conventional adenomas compared to the control samples. These microorganisms have been positively associated with anti-inflammatory diets characterized by a high consumption of vegetables and a reduced risk of CRC; however, they have also been found to be increased in the microbiota of individuals with CRC in several studies [45,46,47]. *Streptococcaceae*, which were also decreased in HGD individuals, are carbohydrate-fermentative and organic acid-producing microorganisms that are commensal inhabitants of the oral cavity and gut; they contribute to immune homeostasis, although some species are pathogenic and have been related to oral disease and CRC [48]. In the present study, the significant decrease in some relevant families of carbohydrate-fermentative microorganisms in the gut (*Christensenellaceae*, *Clostridiaceae*, *Erysipelotrichaceae*, *Streptococcaceae*, *and Prevotellaceae*), together with the decrease in methanogenic archaea, could reflect a progressive alteration of the gut microbial consortia participating in fermentative processes. 

In short, the work performed evidenced deeper changes in the fecal microbiota compositions of individuals with conventional adenomas than in those with hyperplastic polyps. The increased abundance of the *Ruminococcus_torques* group was a differential signature of the microbiota compositions of individuals with polyps, with either conventional adenomas or hyperplastic polyps. The evolution of certain taxonomic groups was different for both types of alterations, and the decrease in specific microbial families as related to the grade of dysplasia in conventional adenomas was of relevance.

Despite the fact that some microbial families were associated with the fecal levels of SCFAs, no significant variations in the fecal concentrations of the major SCFAs were found, which suggests that although important shifts occur in the fermentative gut microbial consortia alongside the progression of intestinal mucosal lesions, they do not globally affect SCFA concentrations. Nevertheless, the relatively small sample size may be influencing the lack of significant differences in this regard. The decrease in the fecal levels of caproic acid in individuals with conventional adenomas with HGD was associated with the decrease in *Oscillospiraceae* amounts in this histopathological group. Caproic acid is mostly derived from diet and can participate in the enhancement of Th1 and Th17 cell differentiation in the immune system [49]. However, the reduced concentration of this compound in feces and the lack of sufficient data preclude our formulating any hypothesis. Alterations to the intestinal microbiota associated with intestinal mucosa damage could be also related to shifts in the concentrations of a variety of metabolites, other than SCFAs, such as those derived from the metabolism of dietary phenolic compounds, amino acids, lipids, nucleotides, and xenobiotics. Metabolomic studies will help to identify the metabolic changes associated with altered microbiota profiles and damage of the intestinal mucosa. 

We have recently reported altered levels of *Lachnospiraceae* and *Eggerthellaceae*, as well as of the *Muribaculaceae*, *Streptococcaceae* and *Eubacterium coprostanoligenes* groups, in a collective of socially vulnerable individuals associated with the consumption of 2-amino-3,8-dimethylimidazo [4,5-f]quinoxaline (MeIQx) and PhIP, which are two heterocyclic amines formed during food cooking [50]. Therefore, although a causality cannot be established, these preliminary data may suggest a possible relationship between initial alterations to the intestinal mucosa derived from toxic dietary compounds and gut intestinal microbiota profiles. 

## 5. Conclusions

The comparison between control and polyps groups and the differences found examining the histopathological features, together with the grade of dysplasia in stages prior to CRC development, revealed important shifts in fecal microbiota profiles; some of the relevant taxa compositions that were altered are microorganisms that are normally involved in fermentative processes in the intestine. 

The present work could provide a starting point for wider studies focused on the possible influence regarding modification of diet and lifestyle on the initial stages of intestinal alterations that could potentially trigger the CRC process and other diseases.

## Figures and Tables

**Table 1:** General characteristics of the sample population according to the diagnosis after colonoscopy and histopathological analysis of intestinal mucosa resected samples during colonoscopy.

Variables	Control (*n* = 20)	Polyps (*n* = 34)
Male gender	6 (30.0%)	19 (55.9%)
Age (years)	60 ± 9	61 ± 6
BMI (kg/m^2^) ^†^	25.67 ± 3.81	27.87 ± 4.16
Histopathological analysis ^††^		
NP	20 (100.0%)	-
Hyperplastic polyps	-	9 (26.5%)
Conventional adenomas	-	25 (73.5%)
* LGD*	-	20
* HGD*	-	5

Values are presented in terms of n (percentage in the sample population) or mean ± standard deviation (SD). No significant differences (*p* ≥ 0.05) were found for gender (chi-squared test), age, and BMI (Mann–Whitney U test) between diagnosis groups. ^†^ The number of volunteers with BMI available information was the following: control: *n* = 14; polyps: *n* = 27. ^††^ Samples from individuals with more than one type of polyp were considered in the histopathological group of that of the highest risk. BMI: body mass index; NP: nonpathological histology; LGD: conventional adenomas with low-grade dysplasia; HGD: conventional adenomas with high-grade dysplasia.

**Table 2:** Fecal short-chain fatty acid (SCFA) concentrations (in mM) according to histopathological groups.

	Histopathological Groups		
Fecal SCFAs (mM)	Control (*n* = 20)	Hyperplastic Polyps (*n* = 9)	Conventional Adenomas (*n* = 25)
Acetic acid	47.16 ± 22.35	37.98 ± 23.88	49.86 ± 23.46
Propionic acid	13.65 ± 8.08	14.10 ± 7.94	15.92 ± 8.42
Butyric acid	13.16 ± 9.73	9.38 ± 6.04	11.48 ± 6.67
Isobutyric acid	0.99 ± 1.11	1.08 ± 1.29	0.96 ± 1.01
Isovaleric acid	2.15 ± 1.85	2.06 ± 1.21	2.18 ± 1.76
Valeric acid	2.38 ± 1.77	1.33 ± 0.37	1.67 ± 1.01
Caproic acid	0.80 ± 1.26	0.17 ± 0.35 *	0.23 ± 0.45 *

Values are presented in terms of mean ± standard deviation. (*) Statistically significant differences of hyperplastic polyps or conventional adenomas compared to control group (Mann–Whitney U test; *p* < 0.05). SCFAs: short chain fatty acids.

**Table 3:** Results obtained from linear regression analyses identifying bacterial families as predictors of SCFAs according to the diagnosis and histopathological groups of volunteers.

Diagnosis Group	Dependent Variable	Independent Variable	R^2^	β	*p*
Control	Acetic acid	*Lachnospiraceae*	0.301	0.581	0.007
	Propionic acid	*Oscillospiraceae*	0.434	−0.623	0.002
		*Coprobacillaceae*	0.434	0.376	0.044
	Butyric acid	*Oscillospiraceae*	0.275	−0.560	0.010
Polyps	Acetic acid	*Oscillospiraceae*	0.240	−0.512	0.002
	Propionic acid	*Rikenellaceae*	0.232	−0.505	0.002
	Butyric acid	*Rikenellaceae*	0.145	−0.413	0.015
	Isobutyric acid	*Enterobacteriaceae*	0.267	0.537	0.001
	Isovaleric acid	*Lachnospiraceae*	0.115	−0.376	0.028
Hyperplastic polyps	Propionic acid	*Rikenellaceae*	0.388	−0.681	0.043
	Butyric acid	*Oscillospiraceae*	0.373	−0.672	0.047
	Isobutyric acid	*Enterobacteriaceae*	0.943	0.975	0.000
	Isovaleric acid	*Enterobacteriaceae*	0.835	0.925	0.000
Conventional adenomas	Acetic acid	*Rikenellaceae*	0.238	−0.519	0.008
	Propionic acid	*Christensenellaceae*	0.312	−0.423	0.022
		*Coprobacillaceae*	0.312	0.367	0.044
	Butyric acid	*Coprobacillaceae*	0.138	0.417	0.038
Low-grade dysplasia	Acetic acid	*Rikenellaceae*	0.221	−0.512	0.021
	Propionic acid	*Eubacterium coprostanoligenes* group	0.494	−0.544	0.04
		*Christensenellaceae*	0.494	−0.457	0.013
	Isobutyric acid	*Ruminococcaceae*	0.232	−0.522	0.018
High-grade dysplasia	Isobutyric acid	*Enterobacteriaceae*	0.706	0.883	0.047
	Caproic acid	*Oscillospiraceae*	0.739	0.897	0.039

Linear regression analyses were adjusted according to diagnosis and histopathological groups. Only the variables with *p* < 0.05 in each model are shown. R^2^: coefficient of multiple determination; β: standardized regression coefficient.

## Data Availability

The datasets generated during metagenomic sequencing of fecal DNA samples were deposited in the NCBI Sequence Read Archive PRJNA994445 (http://www.ncbi.nlm.nih.gov/bioproject/994445).

## Supplementary Materials

The following supporting information can be downloaded at https://www.mdpi.com/article/10.3390/cancers16010104/s1. Table S1: Differences in the relative abundances of microbial phyla in fecal samples from individuals with conventional adenomas according to the grade of dysplasia; Table S2: Differences in the relative abundances of microbial genera, ranked according to their taxonomic family membership, in fecal samples from individuals with conventional adenomas according to the grade of dysplasia; Table S3: Fecal short-chain fatty acids (SCFAs) concentrations (mM) according to diagnosis groups.
