# Supplementary material for: Commensal Fecal Microbiota Profiles Associated with Initial Stages of Intestinal Mucosa Damage: A Pilot Study
Source: Cancers (Basel). 2023 Dec 24;16(1):104. doi: 10.3390/cancers16010104 (PMC10778549; doi:10.3390/cancers16010104)
Supplement: Supplementary file 1 [file cancers-16-00104-s001.zip › cancers-2726888-supplementary.pdf]

**Table S1**. Differences in the relative abundance of microbial phyla in fecal samples from individuals with conventional adenomas according to the grade of dysplasia.

| **Microbial phyla (% abundance)** | **Control (*n*=20)** | **LGD (*n*=20)** | **HGD (*n*=5)** |
|---|---|---|---|
| *Bacillota* | 57.60 ± 11.86 | 53.48 ± 12.27 | 51.47 ± 20.63 |
| *Actinomycetota* | 21.23 ± 11.08 | 29.06 ± 14.44 | 27.09 ± 15.75 |
| *Bacteroidota* | 10.64 ± 7.55 | 10.19 ± 9.7 | 10.76 ± 5.84 |
| *Pseudomonadota* | 5.10 ± 9.10 | 2.42 ± 2.68 | 2.59 ± 2.70 |
| *Verrucomicrobiota* | 1.24 ± 1.72 | 1.3 ± 2.46 | 4.90 ± 7.46 |
| *Euryarchaeota* | 2.11 ± 2.59 a | 1.63 ± 3.19 a,b | 0.26 ± 0.52 b |
| *Acidobacteriota* | 0.41 ± 0.78 | 0.33 ± 0.45 | 0.88 ± 1.23 |
| *Chloroflexota* | 0.38 ± 0.83 | 0.38 ± 0.53 | 0.36 ± 0.39 |
| *Thermodesulfobacteria* | 0.31 ± 0.60 | 0.26 ± 0.43 | 0.17 ± 0.19 |
| *Myxococcota* | 0.17 ± 0.31 | 0.12 ± 0.16 | 0.41 ± 0.55 |
| Others | 0.81 ± 1.43 | 0.83 ± 0.81 | 1.12 ± 1.14 |

Values are shown as mean ± standard deviation. Values in the same row showing different letters present a statistically significant difference of LGD or HGD with respect to the control group (U Mann Whitney test, adjusted by Benjamini-Hochberg; $p < 0.05$). LGD, low grade dysplasia; HGD, high grade dysplasia.

**Table S2.** Differences in the relative abundance of microbial genera, ranked according to their taxonomic family membership, in fecal samples from individuals with conventional adenomas according to the grade of dysplasia.

| **Microbial genera (% Abundance)** | **Control (*n*=20)** | **LGD (*n*=20)** | **HGD (*n*=5)** |
|---|---|---|---|
| *Bifidobacteriaceae:* | | | |
| *Bifidobacterium* | 9.71 ± 10.37 | 14.83 ± 14.05 | 8.09 ± 11.82 |
| *Prevotellaceae:* | | | |
| *Prevotella* | 3.14 ± 6.27 | 3.37 ± 9.07 | 1.61 ± 3.31 |
| *Coriobacteriaceae:* | | | |
| *Collinsella* | 6.33 ± 3.87 | 8.66 ± 6.61 | 12.73 ± 13.41 |
| *Peptostreptococcaceae:* | | | |
| *Intestinibacter* | 2.16 ± 3.65 | 1.53 ± 2.35 | 0.97 ± 1.58 |
| *Romboutsia* | 5.13 ± 4.64 a | 2.73 ± 2.47 b | 3.12 ± 3.65 a,b |
| *Clostridiaceae:* | | | |
| *Clostridium_sensu_stricto_1* | 1.42 ± 1.72 a | 0.68 ± 0.45 b | 1.10 ± 1.76 a,b |
| *Bacteroidaceae:* | | | |
| *Bacteroides* | 3.94 ± 2.65 | 4.56 ± 4.39 | 7.76 ± 6.62 |
| *Enterobacteriaceae:* | | | |
| *Escherichia_Shigella* | 3.04 ± 7.69 | 0.47 ± 0.97 | 0.24 ± 0.30 |
| *Veillonellaceae:* | | | |
| *Dialister* | 0.91 ± 1.02 | 3.22 ± 6.19 | 1.67 ± 1.84 |
| *Lachnospiraceae:* | | | |
| *Agathobacter* | 4.47 ± 3.27 | 6.04 ± 4.81 | 4.18 ± 4.10 |
| *Roseburia* | 1.24 ± 1.15 | 1.11 ± 1.06 | 1.11 ± 0.93 |
| *Blautia* | 2.36 ± 1.12 | 2.51 ± 0.93 | 2.72 ± 1.36 |
| *Fusicatenibacter* | 1.11 ± 1.13 | 1.18 ± 0.64 | 1.02 ± 0.76 |
| *Coprococcus* | 1.25 ± 0.94 | 1.10 ± 0.72 | 0.88 ± 0.59 |
| *Dorea* | 1.17 ± 0.54 | 1.55 ± 0.77 | 1.27 ± 0.96 |
| *Ruminococcus_gnavus* group | 0.27 ± 0.71 b | 0.60 ± 0.89 b | 1.38 ± 2.49 a,b |
| *Ruminococcus_torques* group | 0.62 ± 0.37 b | 0.99 ± 0.60 b | 1.22 ± 1.52 a,b |
| *Akkermansiaceae:* | | | |
| *Akkermansia* | 1.14 ± 1.65 | 1.19 ± 2.46 | 4.65 ± 7.62 |
| *Oscillospiraceae:* | | | |
| *Oscillospiraceae_UCG-002* | 1.64 ± 1.15 | 1.07 ± 1.00 | 0.66 ± 0.56 |
| *Streptococcaceae:* | | | |
| *Streptococcus* | 2.22 ± 3.87 a | 0.98 ± 1.49 a,b | 0.25 ± 0.20 b |
| *Methanobacteriaceae:* | | | |
| *Methanobrevibacter* | 1.99 ± 2.50 a | 1.60 ± 3.13 b | 0.26 ± 0.52 b |
| *Erysipelotrichaceae:* | | | |
| *Holdemanella* | 1.33 ± 1.89 a | 0.68 ± 1.32 b | 0.19 ± 0.23 a,b |
| *Eggerthellaceae:* | | | |
| *Slackia* | 0.92 ± 1.12 | 0.65 ± 0.78 | 1.33 ± 1.50 |
| *Senegalimassilia* | 0.68 ± 0.60 | 1.06 ± 1.05 | 0.61 ± 1.11 |
| *Christensenellaceae:* | | | |
| *Christensenellaceae_R-7* group | 1.86 ± 1.13 a | 1.08 ± 1.16 b | 0.98 ± 1.32 a,b |
| *Ruminococcaceae:* | | | |
| *Subdoligranulum* | 3.60 ± 2.89 | 2.91 ± 2.16 | 3.39 ± 2.15 |
| *Faecalibacterium* | 5.87 ± 2.97 | 4.59 ± 3.12 | 5.12 ± 2.49 |
| *Clostridium* sp. *CAG-352* | 1.07 ± 2.60 a | 0.62 ± 1.56 b | 0.29 ± 0.34 a,b |

| | | | |
|---|---|---|---|
| *Ruminococcus* | 0.54 ± 0.33 a | 0.37 ± 0.3 b | 0.33 ± 0.34 a,b |
| *Rikenellaceae:* | | | |
| *Alistipes* | 0.34 ± 0.31 | 0.51 ± 0.55 | 0.45 ± 0.37 |
| *Eubacterium coprostanoligenes* group: | | | |
| *Eubacterium coprostanoligenes* group | 1.30 ± 0.54 | 1.22 ± 0.66 | 1.12 ± 0.79 |
| *Clostridia_UCG-014* | 1.05 ± 0.87 a | 0.67 ± 1.19 b | 0.63 ± 0.93 a,b |

Values are shown as mean ± standard deviation. Values in the same row showing different letters present a statistically significant difference of LGD or HGD with respect to the control group (U Mann Whitney test, adjusted by Benjamini-Hochberg; $p < 0.05$). LGD, low grade dysplasia; HGD, high grade dysplasia.

**Table S3.** Fecal short chain fatty acids (SCFAs) concentrations (mM) according to diagnosis groups

| | Diagnosis groups | |
|---|---|---|
| Fecal SCFAs (mM) | Control (*n*=20) | Polyps (*n*=34) |
| Acetic acid | 47.16 ± 22.35 | 46.72 ± 23.81 |
| Propionic acid | 13.65 ± 8.08 | 15.44 ± 8.22 |
| Butyric acid | 13.16 ± 9.73 | 10.92 ± 6.49 |
| Isobutyric acid | 0.99 ± 1.11 | 0.99 ± 1.07 |
| Isovaleric acid | 2.15 ± 1.85 | 2.15 ± 1.62 |
| Valeric acid | 2.38 ± 1.77 | 1.58 ± 0.90 |
| Caproic acid | 0.80 ± 1.26 | 0.22 ± 0.42 * |

Values are presented as mean ± standard deviation. (*) Statistically significant differences between groups (U Mann Whitney test; $p < 0.05$). SCFAs, short chain fatty acids.
